# The comparative gene expression concern to the seed pigmentation in maize (*Zea mays* L.)

**DOI:** 10.5808/GI.2020.18.3.e29

**Published:** 2020-09-09

**Authors:** Kyu Jin Sa, Ik-Young Choi, Ju Kyong Lee

**Affiliations:** 1 Department of Applied Plant Sciences, College of Agriculture and Life Sciences, Kangwon National University, Chuncheon 24341, Korea; 2 Department of Agriculture and Life Industry, Kangwon National University, Chuncheon 24341, Korea

**Keywords:** anthocyanin, colored and colorless maize, gene expression, maize, RNA sequence

## Abstract

Maize seed pigmentation is one of the important issue to develop maize seed breeding. The differently gene expression was characterized and compared for three inbred lines, such as the pigment accumulated seed (CM22) and non-pigmented seed (CM5 and CM19) at 10 days after pollination. We obtained a total of 63,870, 82,496, and 54,555 contigs by de novo assembly to identify gene expression in the CM22, CM5, and CM19, respectably. In differentially expressed gene analysis, it was revealed that 7,044 genes were differentially expressed by at least two-fold, with 4,067 upregulated in colored maize inbred lines and 2,977 upregulated in colorless maize inbred lines. Of them,18 genes were included to the anthocyanin biosynthesis pathways, while 15 genes were upregulated in both CM22/5 and CM22/19. Additionally, 37 genes were detected in the metabolic pathway concern to the seed pigmentation by BINs analysis using MAPMAN software. Finally, these differently expressed genes may aid in the research on seed pigmentation in maize breeding programs.

## Introduction

Maize (*Zea mays* L.) is one of the most important crops in the world and is used as food, animal feed, and biofuel. Among the total production of maize in the world, about 20% is used for human consumption, while a high percentage of the rest is used as animal feed [1]. Until now, white and yellow color maize are more popular in the world than colored maize, such as those with black, red, purple, and blue kernel [2-4]. Although most consumers prefer white or yellow maize, colored maize contains phytochemicals and many secondary metabolites, such as phenolic compounds and flavonoids [2]. Thus, there has been increasing demand recently for the development of colored kernel maize because of its anti-cancer, anti-inflammatory, and anti-oxidant traits [5]. Due to such interest in colored maize, Korean maize breeding programs have been focusing on the development of functional purple maize varieties, resulting in the development of some purple waxy maize varieties, such as Miheugchal [6], Sekso2 [7], Heukjinjuchal [8], and Cheongchunchal [9]. Therefore, research into the genes associated with seed pigmentation is an essential step to developing breeding materials that can foster a number of varieties in colored maize breeding. Generally, the pigments of colored maize are determined by flavonoids, mainly anthocyanins, and especially phlobaphenes [2,10,11]. Structural flavonoid genes are responsible for the anthocyanin pigmentation in diverse tissues, such as leaves, stems, anthers, and kernels [12]. Previous researches on anthocyanin pigmentation isolated a number of genes, such as c2 (*colorless2), a1(anthocyaninless1), a2 (anthocyaninless2), chi1 (chalcone flavanone isomerase1), fht1 (flavanone 3-hydroxylase1), pr1 (red aleurone1), bz1 (Bronze1), bz2 (Bronze2)*, and transcription factors, such as *Sn1 (scutellar node color1), r1 (colored1), b1 (colored plant1), c1 (colorless1), p1 (Pericarp color1), pl (purple plant)*, and *pac1 (pale aleurone color1)* in the anthocyanin metabolism pathway of maize [10,13,14].

As mentioned above, many genes are involved in the pathway of anthocyanins. A key means to study the mechanisms of pigmentation of maize seed is to confirm its gene expression level and its functions. Thus, identification of the differential expression genes (DEGs) is crucial in understanding the mechanism of seed pigmentation. Because of recent advances in next-generation sequencing, transcriptome analysis by RNA sequencing (RNA-Seq) allows cost-effective research on both the sequence and transcriptional variations for functional genomic analysis, especially in large and repetitive sequence-rich genomes such as maize [15,16]. The transcriptome is the total set of transcripts among different cells or tissue types, as well as their developmental and physiological stages. An analysis of transcriptome dynamics provides the opportunity to imply the function of unannotated genes, identify critical network hub-related genes, and interpret the cellular processes associated with development [17]. Furthermore, this method can obtain single nucleotide polymorphisms, which are of particular interest when studying allele-specific expression patterns [18]. RNA-Seq for whole-transcriptome researches has been used to identify the spatial and temporal expression patterns of transcriptome in different cells or tissues, along with the development stages in maize [19-23]. A comparison of the transcriptomes associated with seed pigmentation is important in understanding the process of accumulation of anthocyanin in different kinds of colored maize. However, there is still limited information on the seed pigmentation-related gene expression during seed development in maize. This study sequenced and assembled three maize inbred lines (one purple colored, CM22, and two white colored, CM5 and CM19) to perform a comparative expression analysis between colored and colorless maize kernels. The profiling of comparative DEGs and the functional classification by the Gene Ontology (GO) between colored and colorless maize were obtained. This study’s results may then provide valuable information for future studies on colored and colorless maize breeding programs.  

## Methods

### Plant materials

Three waxy maize inbred lines, CM22 (colored; purple), CM5, and CM19 (colorless; white), were grown in a field at the College of Agriculture and Life Science of Kangwon National University located in Chuncheon, Gangwon-do, Korea. Ears of each maize inbred line were covered before silk emergence to prevent contamination from immediate pollen. Because each maize inbred line had different tasseling and silking periods, kernels were generated by selfing in time for the flowering phase of each inbred line. At 10 days after pollination (DAP), the seeds of each inbred line were isolated and transferred into independent tubes before being frozen in liquid nitrogen and stored at –80°C until RNA extraction.

### RNA isolation, cDNA library, and sequencing

The total RNA from three maize inbred lines (CM22, CM5, and CM19) was isolated and purified using the Hybrid-R kit (GeneAll Biotechnology Co., Seoul, Korea). The extracted total RNA were processed to quality control using an Agilent 2100 Bioanalyzer (Agilent Technologies, Santa Clara, CA, USA) for high throughput sequencing library. The total RNA of integrity number values with at least eight were used for the subsequent cDNA synthesis. The cDNA library construction and high-throughput mRNA sequencing using the Illumina sequencing platform HiSeq 2500 (Illumina, San Diego, CA, USA) were conducted at the National Instrumentation Center for Environmental Management (NICEM, Seoul National University, Seoul, Korea). The sequencing was generated with 150 bp × 2 of paired-end reads in accordance with the manufacturer’s protocol. The raw reads were filtered out with the trimming of adaptor nucleotides and low-quality nucleotides (reads containing more than 50% bases with Q-value ≤ 20) using trimmomatic [24]. The high-quality data were assembled by means of the Trinity (v2.4.0) *de novo* assembler software to check for newly expressed genes.

### *De novo* assembly and DEG analysis

The new reference gene set was constructed by de novo assembly using Trinity assembler (v2.4.0) and finalized using Transdecoder (v.3.0.1) and CD-EST (v4.6) software. The gene was annotated by BlastX analysis using NCBI non-redundant protein DB. The DEG analysis between colored and colorless lines were analyzed by mapping to the gene set and comparing the expression level. Also, the data were aligned to the maize transcriptome reference B73 RefGen_v3 (https://www.maizegdb.org/) and were checked using RSEM software (https://deweylab.github.io/RSEM/) to comparing expression and reference derived from *de novo* assembly. The DEGs were calculated upon comparing the log2 fold change of the normalization of data of expressed reads using the Trimmed Mean of M values of the EdgeR v3.22.1 DEG analysis package software (https://bioconductor.org/packages/release/bioc/html/edgeR.html). The expressed genes between colored and colorless lines, which were significantly different statistically, were isolated using the false discovery rate < 0.05 value and log2 fold change value.

### Functional and pathway enrichment analysis of DEGs

For functional annotation clustering, The Database for Annotation, Visualization, and Integrated Discovery (DAVID) Bioinformatics Resources v 6.8 software (https://david.ncifcrf.gov/) for the GO categories of cellular component (CC), biology process (BP), and molecular function (MF) was used. The statistically significant GO terms enrichment analysis for DEGs was conducted with thresholds of p < 0.01 and enrichment gene count > 2 in DAVID software. The enriched BINs of the DEGs were confirmed using PageMan analysis, which employs the ORA_FISHER test (cutoff value = 1.0) [25], whereas colored or colorless upregulated genes were assigned to metabolic pathways through the MapMan tool [26]. 

## Results

### Transcriptome sequencing and comparison to reference RNA sequence

To obtain the transcriptional profile in 10 DAP seeds from colored and colorless maize inbred lines, RNA-Seq for three samples, one colored maize inbred line (CM22) and two colorless maize inbred lines (CM5 and CM19), was accomplished. For the 10 DAP seeds of each colorless and colored maize inbred line, a total of 75,257,086 (CM22) colored line, and 103,139,640 (CM5) and 66,978,958 (CM19) colorless line sequence reads were generated with read lengths of about 0.8, 1.0, and 0.7 billion bp, respectively. After filtering high-quality sequences with a Phred quality score of at least 20 and read length of more than 50 bp in the three maize inbred lines, 71.5% (CM22), 71.2% (CM5), and 71.8% (CM19) high-quality reads were gathered from the raw data (Table 1). For the mapping of each transcriptome in the mentioned colored and colorless maize inbred lines, a comparison was made with reference RNA sequences in a public DB (NCBI) which revealed mapped reads of 60%, 62%, and 62% in the expressed sequence tag (EST) references in CM22, CM5, and CM19, respectively (Table 1). The inbred lines revealed with different transcripts with at least 38% to the reference EST sequence.

### *De novo* assembly and DEGs between colored and colorless maize inbred lines

This study assembled the high-quality reads generated from the CM22, CM5, and CM19 maize inbred lines, and a total of 63,870 (CM22), 82,496 (CM5), and 54,555 (CM19) contigs were collected with average lengths of 498 457, and 518 bp, respectively (Table 2). In comparison with the GenBank data, any new genes among the contigs could not be revealed. To estimate the gene expression of unigenes in the three samples, about 50,000 genes (upwards of 50% of total reference genes) were expressed based on the RPKM (reads per kilobase of transcript per million reads mapped) criteria (over 0.25 value) in each of the CM22, CM5, and CM19 maize inbred lines (Table 3) among a total of 98,696 genes of the reference sequence in the NCBI.

**Fig. 1.**
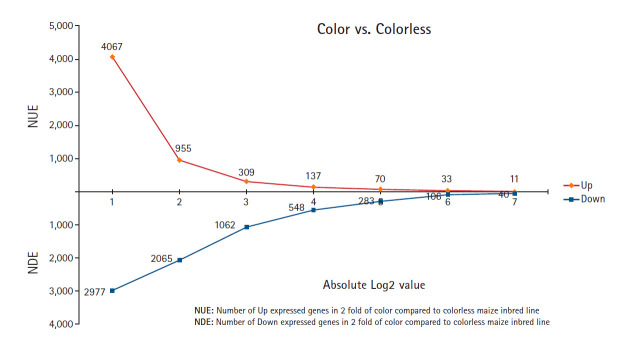
Genes with at least two-fold differences in expression between colored and colorless seed at the development step is presented. A total of 7,044 genes include over two-fold expressed genes. Each value in the X-axis means a log value.

From the DEG analysis, it was revealed that 7,044 genes were differentially expressed by at least two-fold, with 4,067 upregulated in colored maize inbred lines and 2,977 upregulated in colorless maize inbred lines (Fig. 1). This study identified more differentially expressed contigs in colored maize than in colorless maize at different fold change levels: 2–4 fold (3,112 up in colored and 912 up in colorless), 4–8 fold (646 up in colored and 1,003 up in colorless), 8–16 fold (172 up in colored and 514 up in colorless), and ≥16 fold (137 up in colored and 548 up in colorless), indicating changes in expression levels during seed development between colored and colorless maize inbred lines.

### Functional enrichment analysis

A total of 45 GO functions were detected for colored and colorless upregulated genes using DAVID software. The colored upregulated DEGs were enriched in 37 GO terms, while the colorless upregulated DEGs were enriched in 8 GO terms (Table 4). The genes for colored upregulated DEGs were related with enrichments of BP, such as in the anthocyanin-containing compound biosynthetic and metabolic process, carbohydrate metabolic process, cell wall macromolecule metabolic process, cellular carbohydrate metabolic process, cellular glucan metabolic process, cellular polysaccharide metabolic process, flavonoid biosynthetic and metabolic process, glucan metabolic process, photosynthesis-related process, and protein-chromophore linkage; CC, such as apoplast, chloroplast related functions, organelle subcompartment, photosynthetic membrane, photosystem related functions, plastid related functions, and thylakoid related functions; and MF, such as chlorophyll binding, glucosyltransferase activity, glycogen (starch) synthase activity, hydrolase activity related functions, and UDP-glucosyltransferase activity (Table 4). The functional annotation of colorless upregulated DEGs showed trends toward BP, such as response to wounding; MF, such as endopeptidase inhibitor and regulator activity, enzyme inhibitor and regulator activity, and peptidase inhibitor and regulator activity; and serine-type endopeptidase inhibitor activity (Table 4).

### Pathway analysis for colored and colorless maize inbred lines

**Fig. 2.**
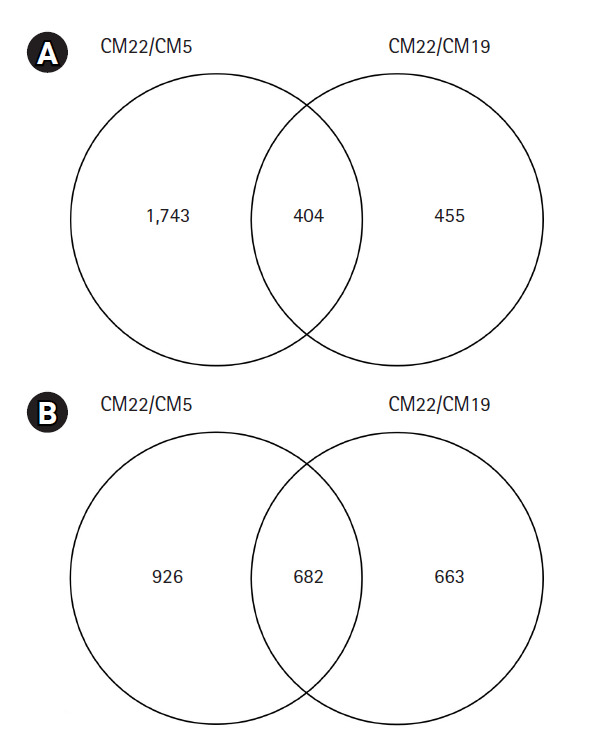
Venn diagram for differentially expressed genes detected in colored and colorless maize inbred lines is presented. (A) Color specific upregulated genes compared with colorless one. (B) Colorless specific upregulated genes compared with colored one.

To reveal specific genes between colored and colorless maize inbred lines, the transcriptional levels of mRNA in two combinations for three maize inbred lines (CM22/CM5 and CM22/CM19) were compared using MapMan software. Among 98,696 genes, a total of 404 genes were upregulated at least over two-fold in CM22 compared with CM5 or CM19 in colored maize inbred lines, whereas a total of 682 genes were upregulated at least over two-fold in CM5 and CM19 compared with CM22 in colorless maize inbred lines (Fig. 2).

**Fig. 3.**
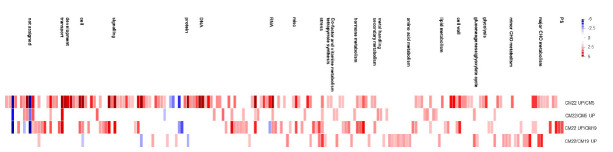
Enriched BINs of differentially expressed genes between colored and colorless maize inbred lines. The box colors represent the average signals of genes involved in each BIN category.

The significant BINs for the representative functional pathways of DEGs were detected in the two combinations of colored and colorless maize inbred lines using Fisher exact test, with a cut-off value of 1.0 employing PageMan analysis (Fig. 3). The enriched BINs of the color upregulated genes were photosynthesis, major and minor CHO metabolism related functions, glycolysis, TCA/org. transformation, mitochondrial electron transport/ATP synthesis, cell wall, lipid metabolism related DEGs, N-metabolism, secondary metabolism, hormone metabolism, tetrapyrrole synthesis, stress, redox.regulation, nucleotide metabolism, miscellaneous related functions, RNA, DNA, protein related functions, signaling, cell, development, transport, and C4.photosynthesis-related DEGs (Fig. 3). In the colorless upregulated genes, there was an overexpression of enriched BINs such as major and minor CHO metabolisms, gluconeogenese/glyoxylate cycle, lipid metabolism, amino acid metabolism related functions, metal handling, secondary metabolism, hormone metabolism related DEGs, co-factor and vitamin metabolism, tetrapyrrole synthesis, stress, miscellaneous related functions, RNA, protein, signaling, and transport-related DEGs.

**Fig. 4.**
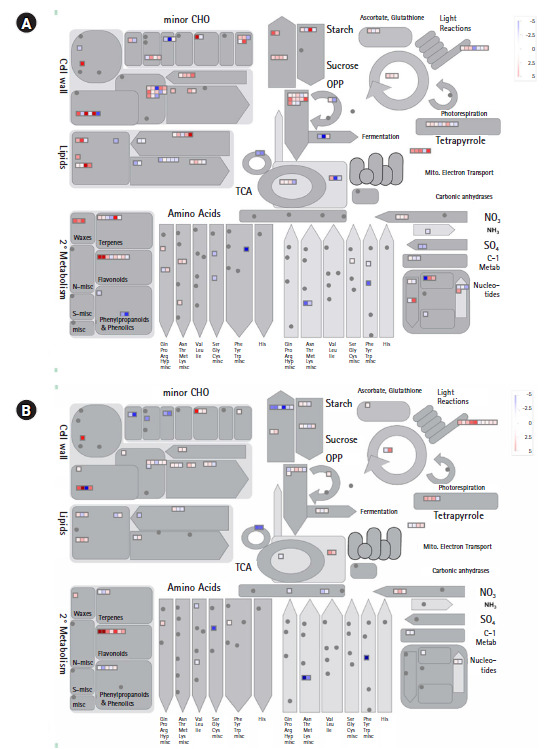
Transcription levels of two-fold differentially expressed genes included in metabolic pathways. (A) CM22/CM5. (B) CM22/CM19.

To confirm expressions of DEGs associated with metabolic pathways, DEGs for each of the two combinations of the three maize inbred lines (based on over two-fold change of expression level for each combination) were assigned to the corresponding BINs using the MAPMAN software (Fig. 4). Among the genes in each of the two combinations (CM22/CM5 and CM22/CM19) of the maize inbred lines, many DEGs in the 10 DAP colored and colorless seeds showed combination-specific (CM22/CM5 or CM22/CM19) expressions in independent metabolic pathways. However, 37 common colored-specific, expressed DEGs in CM22 were detected compared with CM5 and CM19 in metabolic pathways. Although the same genes for each combination had different expression levels, color-specific genes were involved in metabolic pathways (Fig. 4). Meanwhile, 22 common, colorless-specific, expressed DEGs in CM5 and CM19 were confirmed compared with CM22 in metabolic pathways (Fig. 4). 

## Discussion

The comparative analysis of the given colored and colorless maize inbred lines (CM22, CM5, and CM19) was performed to evaluate the DEGs’ profiling between two colored/colorless maize combinations (CM22/CM5 and CM22/CM19). All of the expressed genes were confirmed upon mapping the 98,696 genes of the B73 reference sequences at the NCBI. This study found a number of DEGs by comparing the gene expression between the colored and colorless maize inbred lines. Among the 7,044 DEGs with two-fold elevation in two common maize inbred line combinations (CM22/CM5 and CM22/CM19), 4,067 were upregulated in the colored maize inbred line CM22, while 2,977 were upregulated in the colorless maize inbred lines CM5 and CM19 (Fig. 1).

Based on the GO functional enrichment analysis, the GO terms of upregulated genes for colored or colorless maize inbred lines were differently clustered between color- and colorless-specific, expressed genes. Colored upregulated genes were clustered in many GO terms (Table 4). Photosynthesis-related GO terms had relatively higher functional enrichment score (FES) value (6.53) than other clusters. In previous studies, immature seeds of cereal crop were photosynthetically active in pericarp, which is the outer layer of the seed [27]. In addition to photoreceptors, photosynthesis contributes to the formation of anthocyanin [28]. For example, light-dependent anthocyanin accumulation was significantly inhibited by treatment with a photosynthetic inhibitor in non-chlorophyllous maize leaf [29]. Thus, the differential expression pattern of photosynthesis-related genes in seed pericarp between colored and colorless lines could suggest a dissimilar photosynthesis ability, which may directly affect anthocyanin pigmentation. Moreover, pigmentation-related GO terms were only detected in the third cluster of color upregulated genes that had a 3.37 score for FES (Table 4) while color upregulated genes were clustered in response to wounding, endopeptidase inhibitor and regulator activity, enzyme inhibitor and regulator activity, peptidase inhibitor and regulator activity, and serine-type endopeptidase inhibitor activity (Table 4). These results confirmed that genes directly associated with seed pigmentation only upregulated in colored maize inbred lines (GO:0009812, GO:0009813, GO:0009718, and GO:0046283).

This study also compared gene expression levels for each maize combination, CM22/CM5 and CM22/CM19, with a total of 98,696 maize reference unigenes used for each combination. A total of 1,743 and 455 color-related genes were detected as being upregulated in the colored line CM22 in comparison with each colorless line, CM5 and CM19 (Fig. 2A). Moreover, a total of 926 and 663 colorless related genes were found as upregulated for colorless lines in comparison with the colored line (Fig. 2B). Based on this result, numerous genes upregulated in the colored or colorless line showed a maize inbred specific gene expression pattern. In addition, the CM22/CM19 (upregulated in 455 colored and 663 colorless specific genes) combination had less DEGs than the CM22/CM5 (upregulated in 1,743 colored and 926 colorless specific genes) combination that shared the same genes more because the CM22 inbred line was generated by backcross with CM19.

This study found many DEGs between colored and colorless maize inbred lines (Figs. 1 and 2). Among these DEGs, some colored and colorless upregulated genes with two-fold elevation in two maize combinations showed higher expression in common metabolic pathways and the same function using MapMan based on BINs, such as cell wall.cellulose synthesis//cell wall.modification//glycolysis.glyceraldehyde 3-phosphate dehydrogenase. However, many other genes upregulated in the colored and colorless maize were involved in independent pathways or different functions even in the same pathway (Figs. 3 and 4). These results indicate a difference in the gene function involved in the seed developmental mechanisms for colored and colorless maize that is in agreement with functional enrichment and PageMan analyses (Table 4, Fig. 3).

**Fig. 5.**
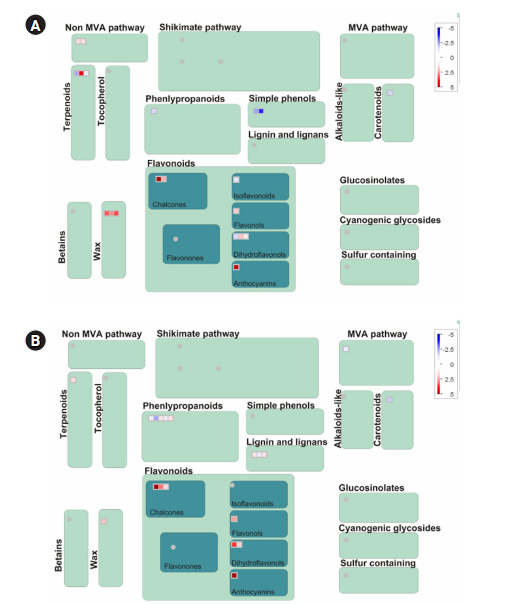
Transcription levels of total genes involved in secondary metabolism pathways in two combinations. (A) CM22/CM5. (B) CM22/CM19.

Seed pigmentation for purple, red, and black colored maize seeds generally occur in the accumulation of anthocyanins. Anthocyanin is synthesized via the flavonoid pathway [30]. Anthocyanin pigmentation in maize organs is controlled by at least 20 loci as structural genes or transcriptional factors [31]. The materials of this study are CM22 with purple color and CM5 and CM19 with white color. Therefore, it is predicted that CM22 had more anthocyanin content and more express gene-related anthocyanin than CM5 and CM19. This study detected the genes, only upregulated in color inbred lines, for the anthocyanin synthesis pathways based on MapMan and gene description (Table 5, Fig. 5). As a result, a total of 18 genes were associated with anthocyanin synthesis in this study. Among these genes, 15 genes were upregulated in both combinations, CM22/CM5 and CM22/CM19, whereas three genes (AF041043, AY103770, and AY108508) were upregulated in only one combination (Table 5). In general, the levels of anthocyanin content increased for color maize during maturation [32]. Moreover, anthocyanin is rapidly accumulated at the later development stages after 25 DAP [30]. The RNA in this study was extracted from the 10 DAP seeds of colored and colorless maize inbred lines. Although many genes upregulated for colored maize inbred lines in this study already have high expression levels, these genes may express themselves more prominently in later development stages.

In this study, the assembled transcriptome method was exploited to perform a comparative expression analysis between one colored and two colorless maize inbred lines. At least a two-fold upregulation of 4,067 genes in colored maize inbred lines and 2,977 genes in colorless maize inbred lines were detected. Although many DEGs were not aligned in a functional pathway by MapMan software, the differences between colored and colorless maize inbred lines were identified in some of the DEGs involved in anthocyanin synthesis. Eventually, these comparative gene expression results may provide valuable information for maize breeding programs.

## Figures and Tables

**Table 1.** Summary of RNA sequencing data, high quality and mapped reads to gene set in the three color and colorless maize inbred lines

Type	Line	Raw sequence data derived from Illumina/HiSeq running		High-quality sequence		Mapped reads to the reference EST	
		Reads	Total length (bp)	Reads	% of total reads	Reads	% of total reads
Color	CM22	75,257,086	7,600,965,686	53,779,012	71.5	44,933,447	60
Colorless	CM5	103,139,640	10,417,103,640	73,476,942	71.2	63,795,970	62
	CM19	6,6978,958	6,764,874,758	48,122,664	71.8	41,301,885	62

EST, expressed sequence tag.

**Table 2.** Contigs by *de novo* assembly in three color and colorless maize inbred lines

Type	Line	Contigs No.	Contigs length	Average length
Color	CM22	63,870	31,799,340	498
Colorless	CM5	82,496	37,678,437	457
	CM19	54,555	28,249,219	518

**Table 3.** Summary of total genes reported in maize and expressed genes with at least 0.25 score normalized with RPKM each maize inbred line

		Total genes	Genes expressed with at least 0.25 score normalized with RPKM	% of total genes
Color	CM22	98,696	51,587	52.3
Colorless	CM5	98,696	53,834	54.5
	CM19	98,696	49,933	50.6

RPKM, reads per kilobase of transcript per million reads mapped.

**Table 4.** Functional enrichment clusters of color and colorless maize genes using the DAVID software

Responses	No. of cluster	FES	Category	GO ID	Term	No. of genes	(%)	p-value
Color-upregulated genes	1	6.53	BP	GO:0009765	Photosynthesis, light harvesting	12	0.99	<0.001
				GO:0018298	Protein-chromophore linkage	13	1.07	<0.001
				GO:0015979	Photosynthesis	25	2.05	<0.001
				GO:0019684	Photosynthesis, light reaction	13	1.07	<0.001
			CC	GO:0009579	Thylakoid	29	2.38	<0.001
				GO:0009521	Photosystem	19	1.56	<0.001
				GO:0034357	Photosynthetic membrane	24	1.97	<0.001
				GO:0009522	Photosystem I	14	1.15	<0.001
				GO:0044436	Thylakoid part	24	1.97	<0.001
				GO:0009523	Photosystem II	16	1.31	<0.001
				GO:0042651	Thylakoid membrane	21	1.73	<0.001
				GO:0055035	Plastid thylakoid membrane	17	1.40	<0.001
				GO:0009535	Chloroplast thylakoid membrane	17	1.40	<0.001
				GO:0009534	Chloroplast thylakoid	17	1.40	<0.001
				GO:0031976	Plastid thylakoid	17	1.40	<0.001
				GO:0044434	Chloroplast part	23	1.89	<0.001
				GO:0044435	Plastid part	23	1.89	<0.001
				GO:0031984	Organelle subcompartment	17	1.40	<0.001
				GO:0009507	Chloroplast	32	2.63	<0.001
				GO:0009536	Plastid	32	2.63	<0.001
			MF	GO:0016168	Chlorophyll binding	12	0.99	<0.001
	2	3.86	BP	GO:0005975	Carbohydrate metabolic process	59	4.85	<0.001
			MF	GO:0016798	Hydrolase activity, acting on glycosyl bonds	36	2.96	<0.001
				GO:0004553	Hydrolase activity, hydrolyzing O-glycosyl compounds	31	2.55	0.001
	3	3.37	BP	GO:0009812	Flavonoid metabolic process	6	0.49	<0.001
				GO:0009813	Flavonoid biosynthetic process	6	0.49	<0.001
				GO:0009718	Anthocyanin-containing compound biosynthetic process	3	0.25	0.006
				GO:0046283	Anthocyanin-containing compound metabolic process	3	0.25	0.006
	4	2.47	BP	GO:0006073	Cellular glucan metabolic process	14	1.15	0.004
				GO:0044042	Glucan metabolic process	14	1.15	0.004
				GO:0044262	Cellular carbohydrate metabolic process	18	1.48	0.005
				GO:0044036	Cell wall macromolecule metabolic process	10	0.82	0.005
				GO:0044264	Cellular polysaccharide metabolic process	14	1.15	0.008
			CC	GO:0048046	Apoplast	8	0.66	0.004
			MF	GO:0004373	Glycogen (starch) synthase activity	5	0.41	0.001
				GO:0035251	UDP-glucosyltransferase activity	10	0.82	0.002
				GO:0046527	Glucosyltransferase activity	10	0.82	0.002
Colorless-upregulated Genes	1	3.84	BP	GO:0009611	Response to wounding	4	0.50	0.005
			MF	GO:0004867	Serine-type endopeptidase inhibitor activity	7	0.87	<0.001
				GO:0004866	Endopeptidase inhibitor activity	8	0.99	<0.001
				GO:0030414	Peptidase inhibitor activity	8	0.99	<0.001
				GO:0061134	Peptidase regulator activity	8	0.99	<0.001
				GO:0061135	Endopeptidase regulator activity	8	0.99	<0.001
				GO:0004857	Enzyme inhibitor activity	12	1.49	<0.001
				GO:0030234	Enzyme regulator activity	12	1.49	0.009

FES, functional enrichment score; BP, biological process; CC, cellular component; MF, molecular function.

**Table 5.** The gene list and expression level involved in anthocyanin related pathway

ID	Gene name	Description	CM22/CM5	CM22/CM19
AF041043	-	Putative dihydroflavonol 4-reductase	1.104	0.097
AM156908	*myb42*	Transcription factor MYB42 (myb42 gene)	2.467	2.503
AY103770	-	Cinnamoyl-CoA reductase	0.180	1.748
AY106481	-	Chalcone-flavanone isomerase	2.210	2.957
AY108508	*chi1*	Chalcone isomerase	-0.056	1.528
AY109395	*chs*	Chalcone synthase	5.471	7.769
AY135018	*pl*	Transcription factor, pl-bol3 allele	5.561	5.561
AY135019	*pl*	Transcription factor, pl-W22 allele	4.903	4.903
DQ335126	*bz*	UDPG-flavonoid 3-oxy glucosyl transferase, bronze-Bz1-McC allele	3.534	3.532
DT641997	-	Oxidoreductase, 2OG-Fe(II) oxygenase	1.777	2.458
EU955769	*C1*	Anthocyanin regulatory C1	1.889	2.439
EU966779	-	Chalcone-flavonone isomerase	5.343	4.404
EU973925	-	Leucoanthocyanidin dioxygenase	2.362	1.945
M26227	*Lc*	Lc regulatory protein	8.076	6.596
U04434	*fht*	Flavanone 3-beta-hydroxylase	1.897	3.592
X15806	*R-S*	Maize anthocyanin regulatory R-S	8.264	7.380
X55314	*a2*	a2 gene	4.638	5.556
X57276	*B-Peru*	B-Peru gene for a regulatory protein	2.454	5.031
